# The Erysipelas of Infants

**Published:** 1856-03

**Authors:** 


*The Erysipelas of Infants.* By M. Hervieux.—The following are the conclusions of a memoir upon this subject recently read by M. Hervieux, at the Société Médicale des Hôpitaux:—1. It is principally observed within the first six weeks. 2. Among the predisposing causes must be mentioned nosocomical influence, epidemic puerperal fever, induration of the cellular tissue, enteritis, changes in the Peyerian glands, gastro-intestinal ramollissement, bronchitis and eruptive fevers which have reached their last stage. 3. The most common determining causes are umbilical suppuration, ulcerative erythema, suppurative inflammation of vaccine pustules, cupping, leeching or blistering, and the too early application of earrings. 4. It seems to be connected with certain affections not only by relations of causality, but also sometimes by those of contiguity. Thus, peritonitis seems to give rise to erysipelas of the abdomen, pleuritis to that of the chest, and ulcerative stomatitis to that of the face. 5. The local phenomena are the same as in the adult, except when it is grafted on sclerema, when the tumefaction presents unusual hardness, and the temperature is lower than normal. 6. The general phenomena are proportioned to its intensity, and especially to its extension. They consist in fever, gradually increasing agitation, sleeplessness, extreme prostration. There is great decoloration of the face, but vomiting or convulsions are rarely present. 7. There is no fever in erysipelas that follows sclerema. 8. Locally, the erysipelas may assume three forms:—the simple, or erythematous, the œdematous, and the bullar. 9. Any part of the body may be occupied by the erysipelas, and the following is the order of frequency:—face, lower part of the trunk and lower limbs, upper limbs, upper part of the trunk, scalp, neck. 10. Sometimes fixed, it is in the majority of cases erratic, this being the form also accompanied by the most intense reaction. 11. Its duration varies from three to eight days. 12. It usually terminates by desquamation, but sometimes by ulceration or gangrene. 23. It is one of the most dangerous diseases of infancy, and the more so the nearer to the period of birth it occurs at. It is less serious when primary than when secondary, when limited than when generalized. 14. The visceral lesions observed are only those found in the diseases which this exanthem complicates. 15. The frightful mortality that takes place in spite of all means employed, calls for great reserve with respect to therapeutical agents.—*Med. Times & Gaz., Jan.* 1856, *from L'Union Méd.* 1855.

